# Task-specific and variability-driven activation of cognitive control processes during motor performance

**DOI:** 10.1038/s41598-018-29007-3

**Published:** 2018-07-17

**Authors:** Christina Stuhr, Charmayne Mary Lee Hughes, Tino Stöckel

**Affiliations:** 1 Sport & Exercise Psychology Unit, Department of Sport Science, University of Rostock, Ulmenstraße 69, 18057 Rostock, Germany; 2 Health Equity Institute, San Francisco, CA 94132 USA; 3 Department of Kinesiology, San Francisco State University, San Francisco, CA 94132 USA

## Abstract

It has long been postulated that cognitive and motor functions are functionally intertwined. While the idea received convincing support from neuroimaging studies providing evidence that motor and cognitive processes draw on common neural mechanisms and resources, findings from behavioral studies are rather inconsistent. The purpose of the present study was to identify and verify key factors that act on the link between cognitive and motor functions. Specifically we investigated whether it is possible to predict motor skills from cognitive functions. While our results support the idea that motor and cognitive functions are functionally intertwined and different motor skills entail distinct cognitive functions, our data also strongly suggest that the impact of cognitive control processes on motor skill proficiency depends on performance variability, i.e. on how challenging a motor task is. Based on these findings, we presume that motor skills activate specific cognitive control processes on two levels: basic processes that are solely related to the type of the motor task, and variability-driven processes that come into play when performance variability is high. For practitioners, these findings call for specific and challenging motor training interventions to directly tap into the to-be-improved cognitive skills and to involve a maximum of cognitive processes.

## Introduction

While the idea that motor skill proficiency and cognitive capacities are connected was first put forward in the early years of the 20^th^ century^1^, it continues to be an area of great focus by researchers to this day^2–6^. The notion that cognitive control processes (and in particular executive functions) are functionally intertwined with motor skill domains that underlie the ability to control goal directed actions has received convincing support from neuroimaging studies demonstrating that motor and cognitive processes draw on common neural mechanisms and resources (e.g., activation of the dorsolateral prefrontal cortex, striatum and neocerebellum)^3,7–9^. However, the strength of this argument is tempered by results from behavioral studies^10,11^, in which motor and cognitive skills are found to be related in some studies but not in others. From this corpus of work, it appears that the interrelation of motor and cognitive processes (namely executive functions) is task-specific and influenced by the novelty and difficulty of the tasks^3,12–15^.

A recent review of 21 studies in developing populations^11^ reported that the relation between motor skill proficiency and cognitive performance is task-specific (instead of being globally intertwined), with correlation values ranging from no correlation/or weak to strong. Specifically, while motor skills that require gross control (e.g., balance, walking, agility, and flexibility) were only weakly associated with cognitive skills, more complex motor skills (e.g., fine motor hand dexterity, precise aiming, and motor planning) were found to be associated to specific (higher-order) cognitive control processes. For example, manual dexterity was associated to inhibitory control and planning and problem solving, but not to working memory^14–16^, and motor planning abilities were associated to working memory (and higher-order executive functioning) but not inhibitory control processes^12,17,18^.

In addition to the evidence suggesting that motor skill components are linked to specific cognitive control processes, a number of studies have indicated that the relationship between these two processes is influenced by the novelty and difficulty of the task^3,19–21^. While an automatized, well-learned response is sufficient to achieve a goal-directed motor action in certain situations (e.g., walking down an empty street), when a task requires one to concentrate on a specific movement feature or the whole movement to successfully perform the motor task (e.g., when learning a new task, when confronted with novel or changing situational constraints, or when performing a difficult/complex task) the individual engages cognitive control processes to assist in the successful performance of the task^3,20–22^. These findings are supported by neuroimaging studies demonstrating that dorsolateral prefrontal cortex activation is positively related to performance on novel tasks and negatively related to performance on well-learned tasks^21–24^, and that only novel and challenging motor tasks activate the dorsolateral prefrontal cortex and the neocerebellum – areas known to be critical for motor and executive functioning^3,4,6,7^. Compared to situations in which (prefrontal cortex and) executive functions are not necessary (e.g., well-learned and simple tasks), motor tasks that require executive control (e.g., novel, complex/difficult tasks) exhibit rather high performance variability (i.e., trial-to-trial variability)^25^. Thus, we argue that the variability in motor performance determines whether, and to what extent, cognitive control processes are needed for successful task performance.

In this study, we sought to isolate main cognitive control processes associated with two omnipresent everyday life motor skills (i.e., balance control and manual dexterity) that differed in their level of control (i.e., gross vs. fine motor abilities). To achieve this aim, forty-eight healthy young adults completed comprehensive testing of the executive functions described by Diamond^4^ (i.e., working memory, inhibitory control, cognitive flexibility, and response planning), single-leg balance control, and manual dexterity. While most of the existing studies have examined potential relations between cognitive and motor functioning in clinical^26–30^ or developing populations^12,15^, we chose to investigate this issue in healthy young adults as we believe that if cognitive and motor functions are indeed intertwined, this association should also be found outside special populations. We were also interested in exploring the influence of performance variability (as a marker of the novelty, complexity/difficulty of a task) on the relation between specific cognitive control processes and motor functioning. We did this by manipulating the level of difficulty of the single-leg balance control task^31^, as difficulty level can be easily adjusted (e.g. by using a balance pad, or performing the task with eyes closed) and the primary outcome measure of the single-leg balance control task (i.e., the mean sway) is highly sensitive to minimal change (compared to the manual dexterity task)^32^.

We hypothesized that manual dexterity (a fine motor skill) would be associated with inhibitory control^15,16^. However, the existing findings regarding the link between single-leg balance control (a gross motor skill) and cognitive skills are inconclusive^15,16^, and as such specific predictions regarding which specific cognitive skills are involved in balance skills are not justified. In addition, based on the notion that cognitive control processes are primarily engaged when a task is novel or difficult^3,19–21^, we also hypothesized that the influence of executive functions on motor skill performance would be linked to motor variability. That is, we expected that cognitive control processes would exert a weaker influence on balance control in individuals with low performance variability and a greater influence on balance control for individuals who exhibit high performance variability.

## Results

Means, standard deviation and performance variability for all measures of interest are presented in Table 1. All statistical analyses were run on data of forty-eight subjects, except for the balancing task for which we had data of forty-six subjects (two participants did not produce valid trials in all three conditions) and for the Flanker task for which we had data of forty-seven subjects (one data set was incomplete). Preliminary analyses were conducted on the executive and motor function measures to check for normality, sphericity (Mauchly test), univariate and multivariate outliers, with no serious violations noted. As females outperformed male participants for both motor function measures (e.g., manual dexterity mean score: M_female_ = 42.03 ± 3.61, M_male_ = 37.81 ± 3.42, *F*(1,47) = 15.64, *p* < 0.001; balance mean sway: M_female_ = 8.97 ± 1.07 mm, M_male_ = 10.44 ± 2.20 mm, *F*(1,45) = 6.28, *p* = 0.02), gender was considered as a covariate in all analyses. Preliminary data analysis did not reveal any systematic differences for age, physical activity, electronic device use and gaming (all |*r*| < 0.17, all *p* > 0.24). To control for problems of multiple significance testing (e.g., false discovery rate) a Benjamini-Hochberg procedure was applied to the data^33^.

**Table 1:** Descriptive statistics (means and standard deviation, SD) for all study measures of interest and individual performance variability (PV) averaged across participants for the balancing tasks.

		**Mean**	**SD**	**PV**
Motor functioning	Balance control, GKS, mean sway (mm)	9.93	2.00	7.35
	eyes open (EO)	7.63	2.28	5.40
	pad (PAD)	9.26	1.86	6.66
	eyes closed (EC)	12.89	4.43	9.98
	Manual dexterity (MD), Purdue Pegboard (no. of items)	39.22	3.99	
	gross MD	40.69	3.90	
	fine MD	37.74	5.01	
Executive functioning	Processing speed, simple reaction time, RT (ms)	255.11	23.58	
	Working memory, Corsi Block (memory span)	5.45	0.65	
	Response inhibition, Hearts & Flowers			
	congruent condition, RT (ms)	321.07	45.00	
	mixed condition, RT (ms)	548.26	77.30	
	mixed condition, accuracy (%)	86.10	5.30	
	Selective attention, Flanker Fish			
	congruent trials in mixed condition, RT (ms)	695.29	121.16	
	mixed condition, RT (ms)	708.78	97.45	
	mixed condition, accuracy (%)	88.74	4.38	
	Set shifting abilities, Wisconsin Card Sorting, WCST_PE_ (%)	11.74	4.67	
	Response shifting abilities, Trail Making Test			
	TMT-A (sec)	16.66	5.17	
	TMT-B (sec)	39.10	13.05	
	Response planning and problem-solving, Tower of London			
	first move time (sec)	15.41	7.48	
	percent success (%)	75.17	14.94	

### Specific cognitive control processes associated with different types of motor skills

Separate multiple regression analyses were employed to isolate main executive functions that constrain balance control (mean sway) and manual dexterity (mean pegboard score), and to study their relative strength when controlling for the other factors. Balance control was significantly predicted by the full model of cognitive control processes (*R*^2^ = 0.49, *F*(7, 37) = 5.12, *p* < 0.001), with response inhibition (H-RT_acc_; *β* = −0.625, *t*(44) = −3.99, *p* < 0.001), response shifting (TMT-B_A_; *β* = 0.302, *t*(44) = 2.42, *p* = 0.02) and set shifting abilities (WCST_PE_; *β* = 0.254, *t*(44) = 1.93, *p* = 0.06) explaining unique portions (39.1%, 9.1% and 6.5% respectively) of balance control variance. Although the ability of the full model to predict manual dexterity performance did not quite achieve the threshold for statistical significance (*R*^2^ = 0.26, *F*(7, 39) = 1.98, *p* = 0.08), set shifting abilities (WCST_PE_; *β* = −0.419, *t*(46) = −2.72, *p* = 0.01), response planning (TOL_time/acc_; *β* = −0.370, *t*(46) = −2.32, *p* = 0.03), and working memory capacity (CBT_mspan_; *β* = −0.366, *t*(46) = −2.10, *p* = 0.04) appear to explain unique portions of the variance in manual dexterity.

### Indirect effects of cognitive processes on balance control mediated by performance variability

Descriptive statistics (means and standard deviation, SD) for all balance measures are displayed in Table 1. Overall, mean postural sway in single-leg stance was 9.93 mm across the three balance conditions. There was a significant main effect of condition, *F*(2, 88) = 8.04, *p* = 0.001, *η*_*p*_^2^ = 0.15, with significantly lower mean sway values for the EO condition compared to the EC (ΔM = 5.26 mm, *p* = 0.001) and PAD conditions (ΔM = 1.63 mm, *p* = 0.001), and lower mean sway values for PAD compared to the EC condition (ΔM = 3.63 mm, *p* < 0.001). These results are congruent with prior literature^31,34,35^ indicating higher postural instability as task difficulty increases. The main effect of condition on performance variability (i.e., mean sway variability) was also significant, *F*(2, 88) = 6.59, *p* = 0.002, *η*_*p*_^2^ = 0.13. Post-hoc analysis indicated lower mean sway variability values for the EO condition, compared to the PAD (ΔM = 1.26 mm, *p* = 0.001) and EC condition (ΔM = 4.58 mm, *p* = 0.001), and for the PAD compared to the EC condition (ΔM = 3.32 mm, *p* = 0.001). The differences in mean sway variability indicate that the single-leg balance control task can be considered as being highly sensitive to changes in task difficulty (or complexity).

**Figure 1:**
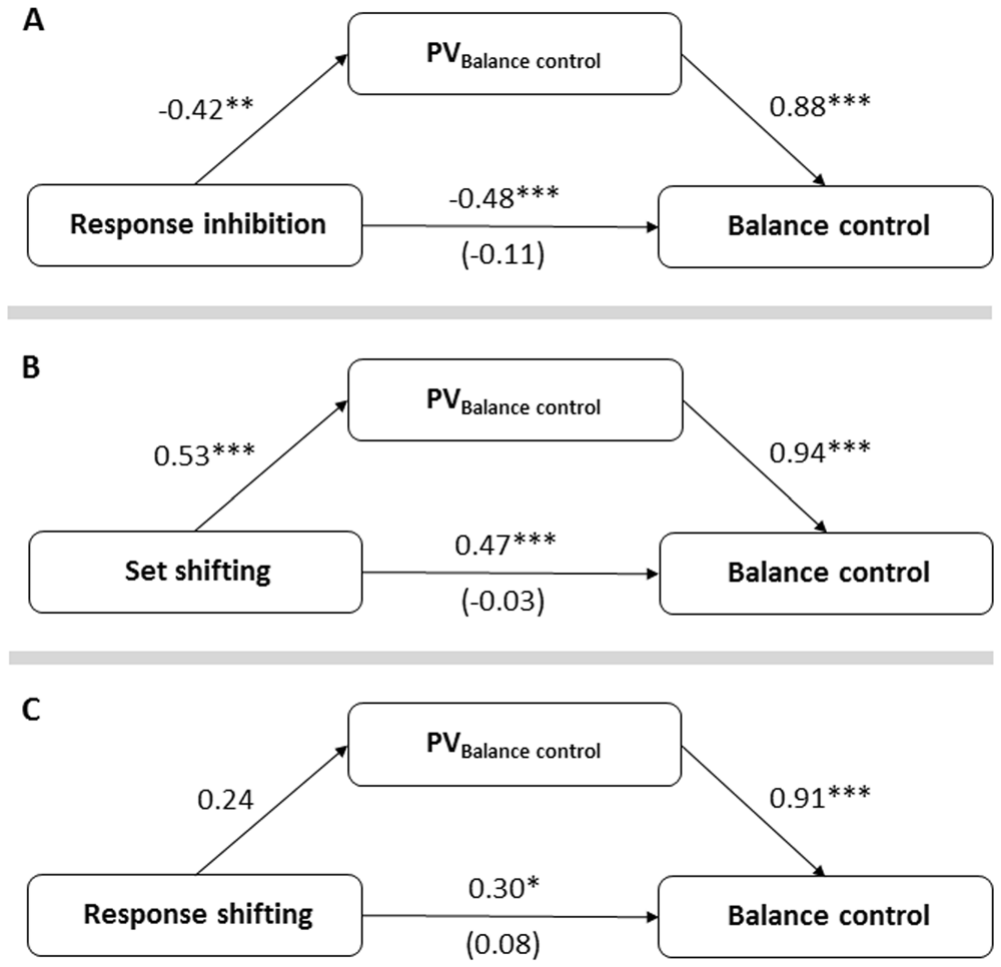
Standardized regression coefficients for the relation between specific cognitive control processes, (**A**) response inhibition, (**B**) set shifting, (**C**) response shifting), and balance control as mediated by performance variability (PV) during one-leg balance. Reported are the standardized regression coefficients along with Benjamini-Hochberg adjusted p-values, ***p < 0.001, **p < 0.01, *p < 0.05, ^§^p < 0.10, N = 46.

Results of the mediation analyses are depicted in Fig. 1. Sobel Z tests revealed significant indirect effects of response inhibition (indirect effect *β* = −0.37; Z = −3.01, *p* = 0.003; Fig. 1A) and set shifting (indirect effect *β* = 0.50; *Z* = 4.13, *p* < 0.001; Fig. 1B) on balance control mediated by performance variability. These effects were confirmed by bootstrapping (based on 5000 samples) for both response inhibition (95% CI: −0.66, −0.07) and set shifting (95% CI: 0.05, 0.85). The results were ambiguous for response shifting: while bootstrapping (based on 5000 samples) yielded an indirect effect of response shifting on balance control mediated by performance variability (95% CI: 0.01, 0.45), Sobel Z test did not confirm this result (indirect effect *β* = 0.22; *Z* = 1.62, *p* = 0.11). Taken together, the relationship between executive and motor functioning is mediated, in large part, by the participants’ performance variability during the single-leg balance control task.

Considering each condition separately, response inhibition (β = −0.36, Z = −2.92, p = 0.04) and set shifting (β = 0.53, Z = 4.47, p < 0.001) exerted a significant indirect effect on balance control during the EC single-leg balance condition, and only response shifting had a significant indirect effect on balance control during the EO balance condition (β = 0.35, Z = −2.68, p = 0.007), while none of the three cognitive control processes had an effect on balance control during the PAD condition. These results support those of the mediation analyses on overall balance control indicating that the association between response shifting and balance control is not mediated by performance variability.

Comparing the total effects (i.e., the sum of direct and indirect effects; see Table 2) between the three balance conditions yields at the strongest associations between cognitive control processes and balance control in the most difficult EC condition for response inhibition (total effect *β* = −0.48, *p* < 0.001) and set shifting (total effect *β* = 0.52, *p* < 0.001).

**Table 2:** Direct effects (i.e., effect of EF on balance control) and indirect effects (i.e., effect of EF on balance control indirectly explained through the mediator, individual performance variability) between the cognitive control processes response inhibition, response shifting and set shifting and balance control averaged across the three conditions and for each balance condition (eyes open, balance pad, eyes closed).

	balance control (average)		eyes open		balance pad		eyes closed	
	*direct*	*indirect*	*direct*	*indirect*	*direct*	*indirect*	*direct*	*indirect*
Response inhibition	−0.11	−0.37**	−0.07	−0.24	0.06	−0.09	−0.13	−0.36**
Response shifting	0.08	0.21	−0.05	0.35**	0.02	0.08	0.07	0.13
Set shifting	−0.03	0.50***	−0.03	0.09	0.001	0.20	−0.002	0.53***

Reported are Sobel test β-values.

Note: ***p < 0.001, **p < 0.01, *p < 0.05 as indicated by Sobel Z test.

## Discussion

The purpose of the present study was to (1) isolate main cognitive control processes associated with two omnipresent everyday life motor skills (i.e., balance control and manual dexterity) that differed in their level of control (i.e., gross vs. fine motor abilities) and (2) explore the influence of performance variability (as a marker of the novelty, complexity or difficulty of a task) on the relation between specific cognitive control processes and motor functioning. In line with findings from neuroimaging studies^3,7,8^, our results support the idea that motor and cognitive functions are intertwined^1,3,6^, as cognitive performance measures explained significant portions of the variance in balance control and also to some extend in manual dexterity. The results of the present study were obtained from healthy young adults who exhibit everyday life motor performance, and as such the link between cognitive and motor processes appears to be quite robust and does not depend on the characteristics of the population (e.g., in children or clinical populations that exhibit impairments in motor and/or cognitive control).

Our data also suggest that (a) different motor skills entail distinct cognitive control processes, and (b) the impact of cognitive control processes on motor skill proficiency is mediated by performance variability (i.e., on how challenging a motor task is), which could explain why empirically reliable evidence for the link between cognitive and motor functioning preferably comes from populations with impaired or limited motor abilities. Measures of executive functioning explained 26% and 49% of the variance in manual dexterity and balance control, respectively. In line with previous reports^12,13,16^, manual dexterity was associated with response planning, working memory and cognitive flexibility, set shifting abilities. By comparison, balance control was associated with response inhibition and both aspects of cognitive flexibility (i.e., set shifting and response shifting abilities).

In contrast to previous reports^15,16,36^, cognitive control processes in general (and inhibitory control in particular) explained large portions of the variance in balance control. Most of the previous investigations^15,16,36^ utilized balance tests from motor batteries which quantify balance performance on a spectrum of time, whereas the primary balance dependent variable (mean sway, in millimeter) used in this study is highly sensitive to small changes in balance control. As such, we argue that the higher sensitivity and validity of the balance measure in the present study helped to identify cognitive control processes associated with balance control. That means that even gross motor skills (such as balance control) require cognitive control processes, and that the type of motor skill (e.g., gross vs. fine) cannot necessarily predict the strength of the relation between motor skill proficiency and cognitive performance, as assumed elsewhere^11^. Moreover, both motor skills were related to set shifting abilities; whereas working memory and response planning were solely related to manual dexterity, and response inhibition and response shifting abilities were solely related to balance control. Taken together, our data strongly suggest that different motor skills entail very specific cognitive control processes^12^.

With regard to the influence of performance variability (as indicator of task difficulty) on the relation between cognitive and motor processes, our data indicated that the relations between the specific cognitive processes and balancing performance are mediated by performance variability, and thus accounted for a large part of the association between cognitive and motor processes. This finding adds nicely to neurobehavioral data indicating that the activation of brain regions critical for executive functioning^3,4,6,7^ are positively associated to the novelty and complexity of a motor task^21–24^ (i.e., task characteristics known to come along with an increased performance variability)^25^. Interestingly, it appears that not all of the cognitive control processes associated to balance control are affected equally by a higher performance variability (i.e., task difficulty). While the influence of set shifting abilities and, to some extent, response inhibition on balance control seems to be strongly associated to performance variability, the link between response shifting abilities and balance control is less affected by performance variability. Specifically, the lack of an association between response shifting abilities and balance control in the eyes closed condition (i.e. the condition with the highest performance variability averaged across all participants) is suggestive of a more global involvement of this cognitive control process in balance control. Based on these findings, we argue that motor skills activate specific cognitive control processes on two levels: (1) *basic cognitive control processes* that are solely related to the type of the motor task (e.g., balancing tasks require response inhibition vs. manual dexterity requires working memory), and (2) *variability-driven cognitive control processes* that come into play when performance variability is high for a given motor task, such as when learning a new motor task, or performing a difficult/complex motor task (e.g., set shifting abilities are required to successfully perform the most challenging aspects of balancing [i.e., eyes closed condition]).

The observation that balance control during the PAD condition was not related to executive function measures is intriguing given that standing on an unstable base of support decreases the reliability of sensory information from plantar mechanoreceptors^37,38^ and creates unexpected mechanical perturbations when compressive loads are applied and released^39^. There is evidence that the visual system is the primary sensory system used to maintain upright postural control^40–42^, and that standing on a foam surface with eyes open leads to an increase in the reliance on visual and/or vestibular inputs to control posture^43,44^, corrective muscle and torque activity^45^, and co-contraction of leg and trunk muscles^46^. Thus, the short-latency postural responses that occur when standing on an instable surface with eyes open is likely to more heavily involve subcortical brainstem and spinal cord structures (e.g., stretch reflex and non-reciprocal inhibition)^47,48^. In contrast, postural control is more likely to involve the cerebral cortex, and associated high-level cognitive processes, when visual input is removed^48^. We speculate that asking participants to stand on a foam surface with eyes closed (i.e., manipulating both surface properties and visual input) would engage both the automatic and cognitive postural control processes, and thus would reveal indirect effect of response inhibition and set shifting on balance control.

Our study comes with some limitations. First, as we employed a cross-sectional design our study neither addressed nor implied causality between measures of motor and cognitive functioning. Future research should employ manipulative designs (e.g., specific motor versus cognitive training interventions of different complexity) in order to test for causality and to identify practice schedules that are most efficient to enhance cognitive and/or motor development. Second, mediation analysis can always only be seen as an indicator of a potential mediation as long as the independent measure has not been systematically manipulated. Therefore, future studies should employ approaches that allow differentiating performance variability between subjects and between tasks. Finally, given the restricted set of motor control tasks being used in the present study, it would be useful to test whether the present findings generalize to different motor skills.

Limitations notwithstanding, our findings provide further support to the principles of executive function training^19,49^. First, transfer of executive function training is very limited to specific motor and cognitive skills. This result may be of use for practitioners who wish to enhance a client’s cognitive performance by means of motor skill training: our findings call for specific motor interventions that directly tap into the to-be-improved cognitive control process. Based on our results, it is very likely that motor tasks involving high balancing demands act more on cognitive processes (e.g., response inhibition or response shifting) that are required to control and optimize ongoing behavior, while motor tasks with manual dexterity demands are more likely to require cognitive processes that assist in the selection of information and advanced planning of actions (e.g., set shifting, working memory or response planning)^13^. Second, motor training interventions need to be challenging to evoke improvements in executive functioning. It is only when a motor task is sufficiently complex/difficult or novel that the training intervention has the potential to involve a large number of cognitive control processes (e.g., set shifting abilities come into play when balancing demands are high). In that regard, constant monitoring of performance variability might help practitioners to maintain or increase the difficulty of the task so that it is challenging at all times. If performance variability is too low (indicative of well-learned or simple tasks for which no or less executive control is required), new situational or environmental constraints should be integrated into the task. As such, practitioners should identify and monitor a client’s cognitive needs and deficits in order to schedule effective training interventions, as global cognitive improvements cannot be expected from motor training.

In conclusion, our data support the idea that motor and cognitive functions are functionally intertwined. The link between the two domains appears to be quite robust, as we found evidence for such a link for highly automatized, omnipresent everyday life motor functions performed by healthy young adults. Our data also adds to the literature by demonstrating that different motor skills entail distinct cognitive control processes, and that the impact of cognitive control processes on motor skill proficiency depends on performance variability (i.e., the level of task difficulty). Based on these findings, we argue that motor skills activate specific cognitive control processes on two levels: 1) basic processes that are solely related to the type of the motor task, and 2) variability-driven processes that come into play when (motor) performance variability is high. For practitioners, these findings call for specific and challenging motor training interventions to directly tap into the to-be-improved cognitive skills and to involve a large number of cognitive processes.

## Materials and Methods

### Participants

Forty eight healthy young adults (age range = 18–35 years, mean age = 23.3 ± 4.3; 32 men) volunteered in this study. On average, participants engaged in 6.7 (±3.5) hours of physical activity (sport) per week, used a personal computer, laptop, smartphone, or tablet 5.0 (±2.7) hours per day and played video games for 1.0 (±1.2) hours per day. Individuals with a history of neurological and/or mental disorders were excluded from the study. The study was approved by the local institutional review board at the University of Rostock and conformed to the declaration of Helsinki. Prior to participation, written informed consent was obtained from all participants.

### Measures and Procedure

All participants completed a comprehensive assessment of motor and cognitive performance using well-established standard tests. The motor skills tested were one-leg balance and manual dexterity. The cognitive control processes tested were working memory, inhibitory control, cognitive flexibility, response planning and problem-solving, and processing speed. Participants were tested individually in four quiet rooms, each of which being specifically equipped to capture a predefined part of the study measures: (1) balance platform to assess one-leg balance (GKS 1000, IMM, Mittweida, Germany); (2) Purdue Pegboard to assess manual dexterity (#32020, Lafayette Instruments, IN, USA)^32^; (3) computerized tests to assess processing speed and inhibitory control using Presentations® (Neurobehavioral Systems Inc., Berkeley, USA) on a 23″ touchscreen monitor (Philips 231C5TJKFU/00); (4) computerized tests to assess working memory, cognitive flexibility, and response planning using the Psychology Experiment Building Language (PEBL)^50^. The task order was randomized across participants. The entire testing lasted approximately two hours.

#### Motor Function Measures

Balance Control: Balance control was assessed using single-leg static balance following recommended test protocols (e.g., warm-up, room temperature, and knee flexion angles)^31,51^. Three single-leg balance conditions were utilized based on their varying difficulty and common use in the literature^52,53^: (1) eyes open on firm ground (EO condition), (2) eyes open on a closed-cell foam elastic balance pad (Airex, Aalen-Ebnat, Germany) (PAD condition), and (3) eyes closed on firm ground (EC condition). It is assumed that single-leg balancing are progressively more difficult in the PAD and EC conditions, compared to the EO condition. These assumptions are grounded in the notion that: (1) reductions of reliable proprioceptive input (i.e., PAD condition) and the elimination of visual input (i.e., EC condition) increase attentional demands for postural control^31,35,54^ and cortical activity in the parietal and central brain areas^55^, and (2) that healthy individuals primarily rely on visual feedback during balance control^35^. Each condition was performed three times (following three familiarization trials) with conditions being block-randomized across participants.

Displacements of the center of pressure (CoP) in the medio-lateral (ML) and anterior-posterior (AP) directions were recorded using a computerized balance platform (GKS 1000, IMM, Mittweida, Germany). Data was collected for 30 seconds at a sample rate of 40 Hz. Postural control for each condition was assessed with the following variables: mean sway (in millimeter) averaged across sway direction and trials; and performance variability (PVbalance control) calculated as the within-trial standard deviation of the COP time series.

Manual Dexterity: Manual dexterity was assessed using the *Purdue Pegboard Test* (#32020, Lafayette Instruments, IN, USA) in which participants were asked to place as many items (pins, washers and collars) as possible on a board with two vertical rows of 25 holes in a predefined order within a given time. The test was administered as outlined in the user instructions^32^. That is, at first participants had 30 seconds to insert as many pins as possible into the holes separately with their right, left and both hands with each condition performed three times. The scores (number of pins inserted) of the three subtasks were combined and used as a measure of gross manual dexterity (MD_gross_). In a second condition participants had 60 seconds to build as many assemblies as possible consisting of a pin, a washer, a collar and another washer using both hands simultaneously and with predefined duties. The score (number of items in completed assemblies plus items in incomplete assemblies averaged across the three trials) was used as a measure of fine manual dexterity (MD_fine_). The score averaged across fine and gross manual dexterity conditions was used as primary measure of manual dexterity.

#### Cognitive Function Measures

Cognitive performance was assessed using a variety of well-established and commonly used standard tests covering the core and higher-level executive functions (as key processes of cognitive control) described by Diamond^4^. The tests on working memory, cognitive flexibility and response planning and problem-solving were built and run using the PEBL software v0.14^50^ (see^56^ for validation). Tests on processing speed and inhibitory control were built and run using Presentations® (Neurobehavioral Systems Inc., Berkeley, USA) along with a 23″ touchscreen monitor (Philips 231C5TJKFU/00) for data input and with hands remaining on a handlebar of 40 cm in length and 5 cm in front of the monitor during the experiments.

Working Memory: The *Corsi Block-Tapping Test*^57,58^ was used as a measure of visuospatial working memory capacity (i.e., the visual-spatial sketchpad)^59^. In this test a set of blue blocks arranged in a static spatial array (on black background) changed color from blue to yellow in a predetermined sequence and participants were asked to reproduce the sequence by tapping on the blocks in the same order they lit up. The test started with three blocks to be tapped and increased by one block up to a maximum of nine blocks every time the participant correctly reproduced the sequence in at least one out of two trials. When two trials of a given span length were failed, the test was discontinued. The maximum sequence length that resulted in correct recall in 50% of the trials was used as primary outcome measure (CBT_mspan_).

Inhibitory Control (self-regulation): *Response inhibition* was assessed using the *Hearts & Flowers Test*^60,61^. In this test participants were sitting in front of a touchscreen, on which either a heart or a flower showed up on the right or the left side of the screen. When a heart appeared participants were asked to tap as fast as possible the button on the same side as the heart (congruent condition), when a flower appeared participants were asked to tap as fast as possible the button on the opposite side of where the flower appeared (incongruent condition). While in the first condition only hearts showed up and in a second condition only flowers showed up, in a third condition hearts and flowers showed up in a random order (mixed condition). For all conditions response accuracy and reaction times for correct responses were computed. Trials faster than 250 ms and slower than two standard deviations above the individual mean were excluded from analysis. Mean reaction time residuals^62^ in the mixed condition (as the most challenging one), controlled for the reaction time in the congruent condition (as an indicator of task-specific processing speed) and response accuracy in the mixed condition, were used as measure of response inhibition (H-RT_acc_).

*Selective attention* (also defined as interference control) was measured using a *Flanker Test* using fish instead of arrows^63^. In this test five fish arranged in a horizontal array were presented on the touchscreen monitor. In a first condition, participants were asked to respond as quickly as possible to the direction the central (blue) fish was looking to (ignoring the flanking fish) by pressing the right or left buttons on the touchscreen (classic condition). In a second condition, participants were asked to respond as quickly as possible to the direction the (pink) outside fish were looking to (ignoring the fish in between) by pressing the right or left buttons on the touchscreen (reversed condition). In a third condition, pink and blue fish showed up in a random order and participants were asked to respond as quickly as possible to the direction either the central (when fish were blue) or the outside fish (when fish were pink) were looking to (mixed condition). Each condition comprised congruent trials (flanking and target fish looking in the same direction), incongruent trials (flanking and target fish looking in different directions) and two types of neutral trials (no flanking fish, target fish surrounded by fish looking up or down). For all conditions and sub-conditions response accuracy and reaction times for correct responses were computed. Trials faster than 250 ms and slower than two standard deviations above the individual mean were excluded from analysis. Mean reaction time residuals^62^ in the mixed condition (as the most challenging one), controlled for the reaction time in congruent trials of the mixed condition (as an indicator of task-specific processing speed) and response accuracy in the mixed condition, were used as measure of selective attention (F-RT_acc_).

Cognitive Flexibility: The *Wisconsin Card Sorting Test* (*WCST*) was used to assess participants’ cognitive flexibility^64^, more specifically their *set shifting* abilities^65^. In this test, participants were asked to sort a total of 128 cards onto one of four piles of stimulus cards by matching the color, the shape or the number of symbols on the cards. Participants were not informed about the classification rule, but they received feedback after each trial whether or not the respective card was classified according to the current rule (“correct” or “incorrect”). After ten consecutive cards had been sorted correctly the classification rule changed without warning and participants had to figure out the new rule as quickly as possible. The percentage of perseverative errors (i.e., errors in which the participant used the same rule as in the previous trial) was used as a measure of participants’ ability to flexibly adapt to a new rule and give up an old rule (WCST_PE_).

The *Trail Making Test* (TMT)^66,67^ was also used as measure of cognitive flexibility tapping, however, more into participants’ *response shifting* abilities^65,68^. In part A, participants were asked to connect 25 numbers in ascending order as quickly as possible. In part B, participants were asked to connect 25 numbers (in ascending order) and letters (in alphabetical order) in an alternating fashion (i.e. 1-A-2-B-3-C, etc.). Testing procedure and trouble-shooting followed the descriptions by Bowie and Harvey^66^. The time to complete part B controlled for time to complete part A (as indicator of task-specific processing speed) was used as measure of response shifting (TMT-B_A_).

Higher-level Executive Functions: The *Tower of London task* was administered to assess participants’ *response planning and problem-solving* abilities^69,70^ as critical aspects of higher-level executive functioning^4^. Participants were asked to rearrange a pile of disks from their original configuration (shown in the middle of the computer screen) to match a target configuration (shown at the top left side of the computer screen) in as few steps as possible. The stimuli were based on the standard set of 12 problems^70^ that consisted of 3 disks and constrained pile heights (1, 2, 3), and the rules that only one disk could be moved at a time and disks could not be moved onto a pile that has no more room. Mean first move time (i.e., planning time needed from presentation of the problem until first move) controlled for the percentage of successful trials (i.e., trials solved in the minimum number of moves) was used as measure of participants’ response planning and problem-solving abilities (TOL_time/acc_).

Processing Speed: A simple reaction time test was administered to assess participants’ processing speed. In this test participants were asked to respond as quickly as possible to a stimuli (a red Dinosaur)^71^ presented in the middle of the touchscreen monitor 500 to 2500 ms following a black fixation cross by tapping on the left mouse button with their right index finger. Mean reaction time (averaged across 32 attempts) was used as measure of simple processing speed (S-RT).

### Data analysis

In the first step of analysis, task-specific relations between motor skill proficiency and cognitive performance were examined by employing separate multiple regression analyses for manual dexterity (mean score) and balance control (mean sway). Potential predictors of the two motor skill components included the following z-standardized measures of cognitive control processes that represent the core and higher-order executive functions outlined by Diamond^4^: simple RT, Corsi memory span, RT during the mixed condition of the Hearts & Flowers Test [controlled for accuracy and RT during the congruent condition], RT during the mixed condition of the Flanker Test [controlled for accuracy and RT during the congruent trials within that condition], percent perseverative errors during the Wisconsin Card Sorting Test, the time required to complete part B of the Trail Making Test [controlled for the time required to complete part A], and first move time during the Tower of London Test [controlled for accuracy]).

In the second step, we employed separate mediation analyses for all cognitive control processes that were identified as predictors of balance control (mean sway) in step 1 in order to better understand the role of performance variability (i.e., mean sway variability; an indicator of task difficulty) on the relation between motor and cognitive skills. The primary outcome measure used was mean sway, the independent measures were the cognitive control processes associated with balance control identified in step 1, and the potential mediator was mean sway variability (i.e., performance variability averaged across the three balance control tasks). Sobel Z tests and bias corrected 95% confidence intervals (95% CI; bootstrapping, m = 5000 samples) were used to identify the indirect effects of executive control processes on motor functioning mediated by performance variability. Analyses were ran using Hayes’ PROCESS macro for SPSS^72^.

In the third and final step, direct and indirect effects for each of the three conditions of the balancing task (see Table 2) were analysed in order to determine whether the relation between executive and motor functions is stronger in balancing conditions with a higher PV (i.e. indicative of a higher difficulty or complexity of the task).
